# Factors and Outcomes Attributable to Weaning and Decannulation from Ventilation after Tracheostomy

**DOI:** 10.31662/jmaj.2024-0439

**Published:** 2025-06-06

**Authors:** Ichita Kinoshita, Masaaki Higashino, Masataka Taniuchi, Tetsuya Terada, Takeshi Tochizawa, Ryo Kawata, Shin-ichi Haginomori

**Affiliations:** 1 Department of Otorhinolaryngology-Head and Neck Surgery, Osaka Medical and Pharmaceutical University, Osaka, Japan; 2 Department of Institutional Research, Osaka Medical and Pharmaceutical University, Osaka, Japan

**Keywords:** tracheostomy, mechanical ventilation, decannulation, lymphopenia

## Abstract

**Introduction:**

The goal of tracheostomy in patients with long-term tracheal intubation is to facilitate weaning from mechanical ventilation (MV), achieve decannulation, and ultimately enable discharge to home. In this study, we investigated the factors influencing withdrawal from MV after tracheostomy and cannulation in patients undergoing long-term tracheal intubation. We also examined tracheostomy status (whether the tracheostomy tube was removed and whether the patient was weaned from MV) and discharge outcomes.

**Methods:**

A total of 199 patients who underwent tracheostomy following long-term tracheal intubation were analyzed. Patients were classified into 3 groups based on tracheostomy status: group A (tracheostomy tube removed; n = 35); group B (tracheostomy tube not removed despite weaning from MV; n = 76); and group C (weaning from MV not achieved; n = 88).

**Results:**

The duration of intensive care unit stay did not differ significantly among the groups. However, hospital stay was significantly shorter in group C than in groups A and B. Additionally, the rate of discharge to home was significantly higher in group A. Factors associated with difficulty in weaning from MV included a blood lymphocyte count <500, the presence of chest and abdominal disease, and a body mass index ≥30. Factors contributing to with difficulty in decannulation after weaning from MV included head and neck disease, age ≥75 years, C-Reactive Protein ≥5, and a blood lymphocyte count <500.

**Conclusions:**

Weaning from MV and decannulation are key factors influencing discharge to home in patients undergoing tracheostomy after long-term intubation. These factors are primarily affected by the patient’s underlying disease and general condition. We believe that addressing these factors through nutritional management, rehabilitation, and other supportive measures can improve the quality of life and increase the likelihood of discharge to home.

## Introduction

Tracheostomy is an important surgical technique in airway management. Prolonged mechanical ventilation (MV) after transoral tracheal intubation increases the risk of reduced overall survival, poor functional status, decreasing quality of life, a higher likelihood of medical complications, and greater utilization of healthcare resources ^(1)^. Therefore, tracheostomy is considered when a tracheal intubation period of >1 to 2 weeks is required ^(2)^. The advantages of tracheostomy during long-term tracheal intubation include the ability to discontinue oral endotracheal intubation, eliminate sedative administration, and facilitate the easier management of airway secretions. However, tracheostomy does not necessarily result in weaning from MV. Furthermore, weaning from MV does not always lead to decannulation or vocalization.

The goal of long-term intubation is to discharge the patient home. Ideally, after tracheostomy, the patient should be weaned from MV and undergo decannulation. In this study, we investigated the factors associated with weaning from MV and decannulation after tracheostomy in adult cases of long-term tracheal intubation, along with the outcomes following discharge. We focused on the period from tracheostomy to discharge, with particular emphasis on weaning from MV and subsequent decannulation, and analyzed the relationship of these factors with post-discharge outcomes. We believe this study will provide valuable insights into predicting MV withdrawal and decannulation challenges before tracheostomy in adults. Such predictive capabilities will enable early intervention to address potential issues related to discharge planning and facilitate explanations to the patient and family.

## Materials and Methods

### Patients

A total of 500 adult patients who underwent tracheostomy at our hospital between 2014 and 2019 were included. Of these, tracheostomy was performed for long-term transoral tracheal intubation, and follow-up after discharge was possible for 200 patients. Cases of long-term tracheal intubation were defined as those intubated for >2 weeks or estimated to have been intubated for > 2 weeks. One patient who underwent aspiration prevention surgery following tracheostomy was excluded. There were no coronavirus disease 2019 (COVID-19) patients in this study.

We retrospectively reviewed the medical records of the patients and classified them into 3 groups according to their status after tracheostomy at the point of discharge from our hospital: group A (tracheostomy tube removal and tracheal stoma closure; n = 35), group B (tracheostomy tube removal was not possible after MV weaning; n = 76), and group C (MV weaning was not possible; n = 88). We considered the following parameters: age (years) (20-39, 40-64, 65-74, ≥75), sex (male, female), body mass index [BMI, kg/m^2^] (<18.5, 18.5≤ <25, 25≤ <30, ≥30), smoking history (none, yes), albumin (Alb, g/dL) (≤1.9, 2-2.4, ≥2.5), C-Reactive Protein (CRP, mg/dL) (<5.0, ≥5.0), blood lymphocyte count (cells/μL) (<500, 500-799, ≥800), primary disease (head and neck, chest and abdomen, other), duration from tracheal intubation to tracheostomy (days) (≤7, 8-14, ≥15), and surgical technique (surgical tracheostomy [ST], percutaneous dilatational tracheostomy [PDT]). Blood tests were based on data obtained just before the tracheostomy.

At our hospital, ST was performed by the otolaryngology department in cases where airway access was either challenging or when the primary department had limited experience with tracheostomy procedures. Conversely, in cases with easy airway access, either PDT was performed by intensive care unit (ICU) doctors, or ST was conducted by doctors from the primary department.

### Length of ICU or hospital stay and outcome at discharge

The duration of the ICU and hospital stays after tracheostomy were examined in groups A, B, and C. Additionally, the outcome at discharge (discharge due to death, transfer to another hospital, or discharge to home) was examined. We investigated the extent to which the ability to wean off MV and the possibility of cannulation affected the goal of discharge to home.

### Factors affecting decannulation after weaning from MV

Being able to remove the tracheal cannula at the time of discharge reduces the burden on the patient. Therefore, to examine the factors affecting decannulation after weaning from MV, we compared 35 patients in group A who were decannulated with 76 patients in group B who could not be decannulated after weaning from MV.

### Factors for weaning from MV

MV must be discontinued for a patient to be decannulated. Therefore, to examine the factors affecting weaning from MV, 111 patients (groups A and B) who were weaned from MV were compared with 88 patients in group C who could not be weaned from MV.

### Statistical analysis

Comparisons of the length of ICU/hospital stay between groups A, B, and C were performed using the Bonferroni test. The outcomes at discharge (discharge due to death, transfer to another hospital, or discharge to home) were calculated for the 3 groups and compared using Fisher’s exact test. Fisher’s exact test was also used for univariate analyses to examine factors affecting decannulation after weaning from MV and factors affecting weaning from MV. The evaluated variables included age, sex, BMI, smoking history, Alb, CRP, blood lymphocyte count, primary disease, duration from tracheal intubation to tracheostomy, and surgical technique. Subsequently, logistic regression analysis was applied for the univariable and multivariable analysis of factors. In the multivariable analysis of factors affecting decannulation after weaning from MV, we selected variables based on previous studies ^(3), (4)^ and clinically relevant factors. In the logistic regression analysis, the reference categories were defined as follows: age 20-39 years, female sex, BMI 18.5≤ <25 kg/m^2^, no smoking history, Alb ≤1.9 g/dL, CRP <5.0 mg/dL, blood lymphocyte count <500/μL, primary disease located in the chest and abdomen, and duration from tracheal intubation to tracheostomy ≤7 days. The reference surgical technique was ST. In all analyses, p-values of <0.05 were considered statistically significant.

We used IBM SPSS version 25 for statistical analysis.

## Results

### Length of ICU and hospital stay and outcome at discharge

**Figure 1.**
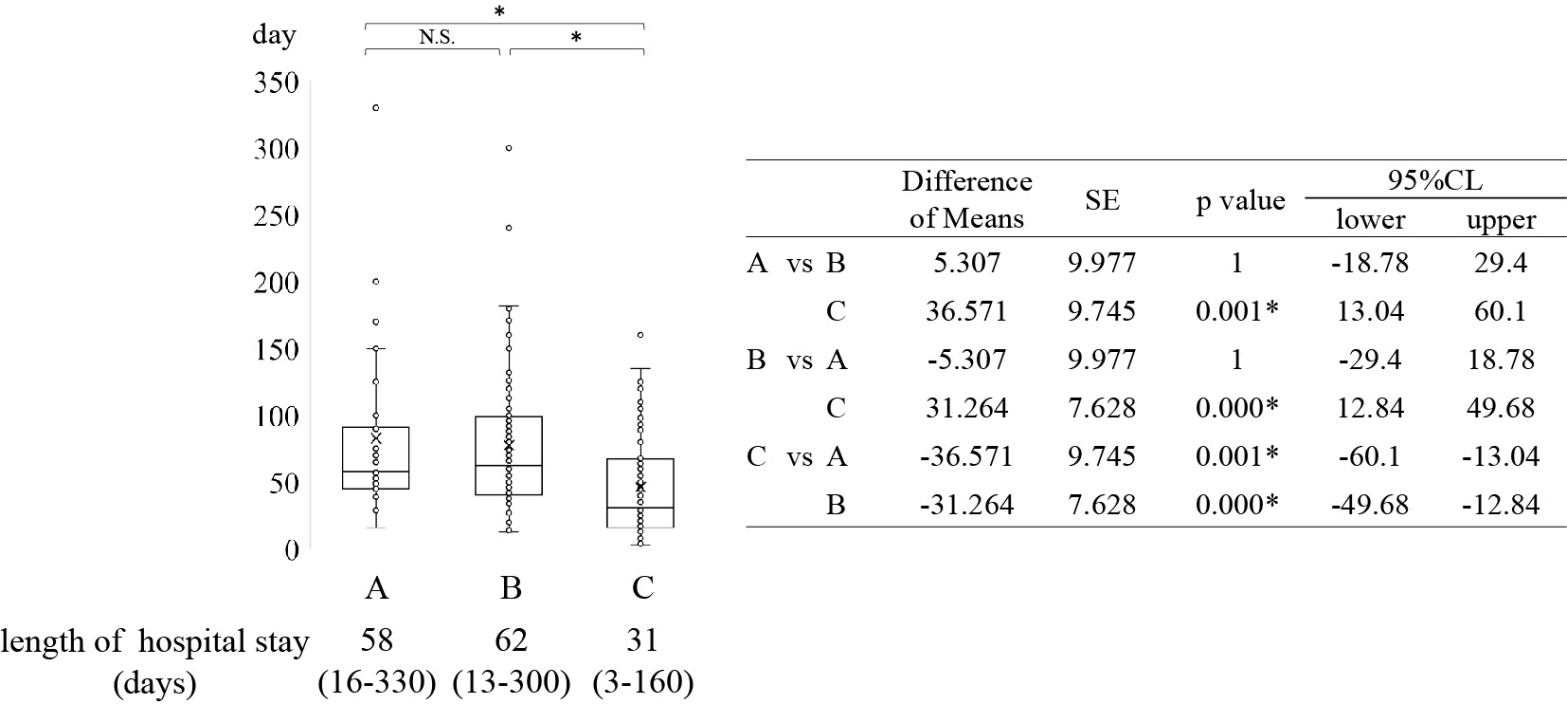
Tracheostomy status and duration of hospitalization after tracheostomy. The duration of hospitalization after tracheostomy was significantly shorter in group A than in group B and C. (*p ≤ 0.001) CI: confidence interval; SE: standard error.

**Figure 2.**
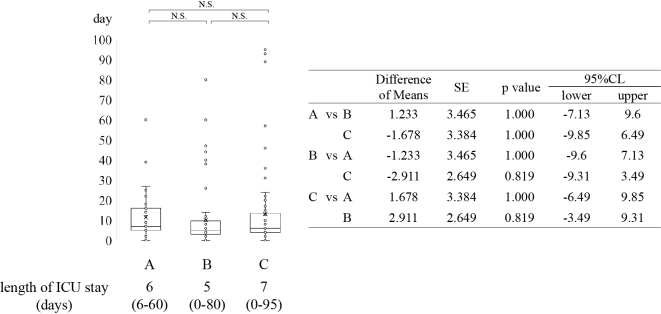
Tracheostomy status and duration of ICU stay. The duration of ICU stay was not significantly different among the 3 groups. CI: confidence interval; ICU: intensive care unit; SE: standard error.

The median length of hospital stay after tracheostomy was 58 days (16-330 days), 62 days (13-300 days), and 31 days (3-160 days) in groups A, B, and C, respectively. Hospital stay was significantly shorter in group C than in groups A and B (Figure 1). The median lengths of ICU stay were 6 days (0-60), 5 days (0-80), and 7 days (0-95) days in groups A, B, and C, respectively (Figure 2).

**Figure 3.**
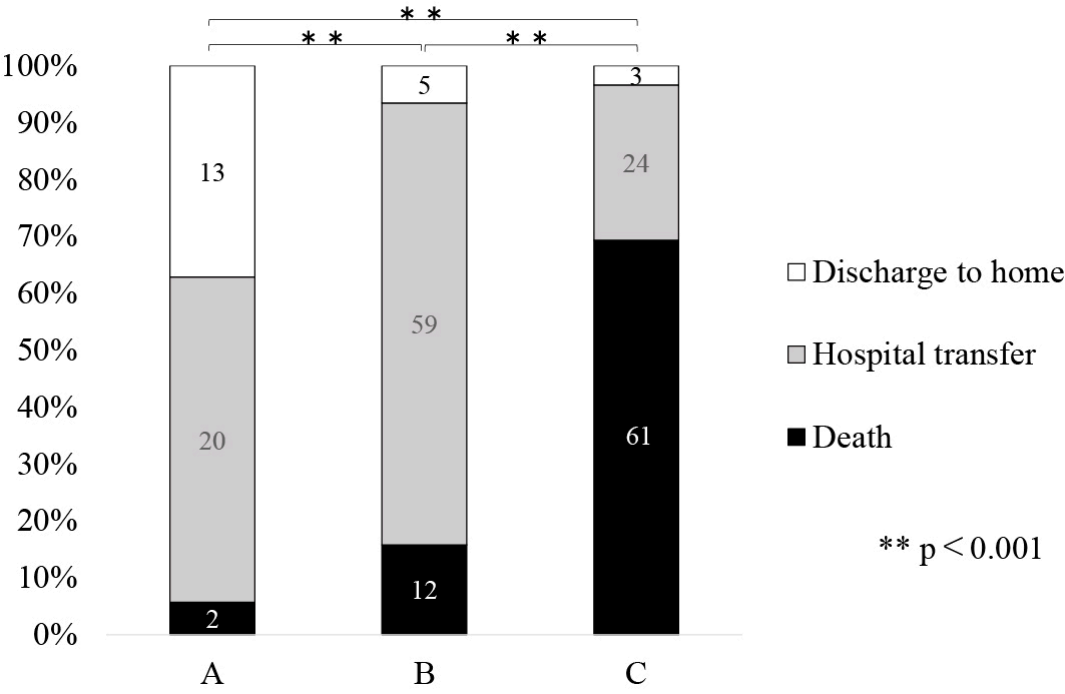
Tracheostomy and patients’ outcomes. There was a significant association between tracheostomy status and patient outcome (**p ≤ 0.001).

Regarding post-discharge outcomes, in group A (n = 35), 13 patients (37.1%) were discharged, 20 patients (57.1%) were transferred to another hospital, and 2 patients (5.7%) died. In group B, 5 patients (6.6%) were discharged, 59 patients (77.6%) were transferred to another hospital, and 12 patients (15.8%) died. In group C, 3 patients (3.4%) were discharged, 24 patients (27.3%) were transferred to another hospital, and 61 patients (69.3%) died. Relative to the other groups, the proportion of discharged patients was significantly higher in group A, the proportion of patients transferred to another hospital was significantly higher in group B, and mortality was significantly higher in group C (*p* < 0.001) (Figure 3). Out of the 21 patients, 13 discharged to home were in group A, suggesting that decannulation is a key factor in achieving the goal of discharge to home.

When classified by patient outcome, 61 of the 75 patients (81.3%) discharged due to death were cases with difficulty weaning from MV. Among the 103 patients transferred to other hospitals, 59 (57.3%) experienced difficulty with cannulation after MV withdrawal. Of the 21 patients discharged home, 13 (61.9%) could be successfully decannulated.

### Factors affecting decannulation after weaning from MV

Comparison occurred between 35 cases in group A and 76 patients in group B. In the univariate analysis, the following factors were significantly more common in group B than in group A: older age (p = 0.01), elevated CRP (p = 0.01), and the presence of head and neck disease (p = 0.01) (Table 1). A multivariable analysis was conducted to identify independent factors. It was significantly more difficult to perform decannulation in patients with head and neck disease and other comorbidities, those aged ≥75 years, those with CRP levels ≥5.0, and those with blood lymphocyte counts <500. This was in comparison to patients with chest and abdominal disease (p < 0.001 and p = 0.009, respectively), patients aged ≤39 years (p = 0.002), patients with CRP <5.0 (p = 0.010), and patients with lymphocytes ≥800 (p = 0.018) (Table 2).

**Table 1.** Comparison of Factors between Patients Who Could or Could Not Be Decannulated.

	Group A (n=35) Successful decannulation	Group B (n=76) Failed decannulation	p value
Age (years) (20-39 : 40-64 : 65-74 : ≥75)	4 : 14 : 8 : 9	3 : 14 : 17 : 42	0.01*
Sex (male : female)	19 : 16	51 : 25	0.28
BMI (kg/m^2^) (<18.5 : 18.5≤ <25 : 25≤ <30 : ≥30)	11 : 19 : 2 : 3	19 : 41 : 13 : 3	0.30
Smoking (No : Yes)	20 : 15	38 : 38	0.62
Albumin (g/dl) (≤1.9 : 2.0-2.4 : ≥2.5)	10 : 12 : 13	25 : 29 : 22	0.69
CRP (mg/dl) (<5.0 : ≥5.0)	25 : 10	33 : 43	0.01*
Lymphocyte Count (/μl) (<500 : 500-799 : ≥800)	4 : 10 : 21	22 : 21 : 33	0.11
Cause of intubation (Head and Neck : Chest and Abdomen : others)	5 : 20 : 10	33 : 28 : 15	0.01*
Intubation period (days) (≤7 : 8-14 : ≥15)	15 : 11 : 9	26 : 25 : 25	0.64
Surgical method (ST : PDT)	17 : 18	52 : 24	0.07

PDT: percutaneous dilatational tracheostomy; ST: surgical tracheostomy.

**Table 2.** Multivariable Analysis of Factors Associated with Decannulation.

		Univariable				Multivariable			
		coef	OR	95%CI	P value	coef	OR	95%CI	P value
Age(year)	20-39	reference				reference			
	40-64	-0.288	0.750	0.141-3.985	0.736	-0.252	0.777	0.106-5.688	0.804
	65-74	-1.041	0.353	0.063-1.964	0.234	-1.452	0.234	0.025-2.172	0.201
	≥75	-1.828	0.161	0.031-0.846	0.031*	-3.937	0.020	0.002-0.240	0.002*
CRP	<5.0	reference				reference			
	≥5.0	-1.181	0.307	0.130-0.727	0.007*	-2.155	0.116	0.022-0.600	0.010*
Lymphocyte count	<500	reference				reference			
	500-799	0.963	2.619	0.710-9.655	0.148	1.099	3.001	0.603-14.941	0.180
	≥800	1.253	3.500	1.057-11.593	0.040*	1.785	5.957	1.358-26.124	0.018*
Cause of intubation	Chest and Abdomen	reference				reference			
	Head and Neck	-1.551	0.212	0.070-0.638	0.006*	-4.192	0.015	0.002-0.130	0.000*
	Others	-0.069	0.933	0.349-2.498	0.891	-2.485	0.083	0.013-0.540	0.009*

CI: confidence interval; coef, coefficient; OR: odds ratio; PDT: percutaneous dilatational tracheostomy; ST: surgical tracheostomy.

### Factors for weaning from MV

Here, a comparison occurred between 88 cases in group C and 111 patients in groups A and B. The univariate analysis revealed that group C had significantly higher rates of older age (p = 0.04), history of smoking (p = 0.02), low serum Alb levels (p = 0.04), presence of chest and abdominal disease (p = 0.004), and lymphopenia (p = 0.006) compared to groups A and B (Table 3). A multivariable analysis was then performed to identify independent factors. It was significantly more difficult to wean patients from MV with a blood lymphocyte count <500, chest and abdominal disease, or BMI ≥30 than those with a lymphocyte count of ≥800 (p = 0.003), patients with head and neck disease (p = 0.003), and patients with BMI 18.5≤ <25 (p = 0.009) (Table 4).

**Table 3.** Comparison of Factors between Patients Who Could or Could Not be Weaned from MV.

	Group A+B (n=111) Successfully weaned	Group C (n=88) Failed to wean	P value
Age (years) (20-39 : 40-64 : 65-74 : ≥75)	7 : 28 : 25 : 51	3 : 13 : 30 : 42	0.04*
Sex (male : female)	70 : 41	64 : 24	0.27
BMI (kg/m^2^) (<18.5 : 18.5≤ <25 : 25≤ <30 : ≥30)	30 : 60 : 15 : 6	25 : 42 : 12 : 9	0.59
Smoking (No : Yes)	58 : 53	29 : 59	0.02*
Albumin (g/dl) (≤1.9 : 2.0≤ <2.4 : ≥2.5)	35 : 41 : 35	37 : 37 : 14	0.04*
CRP (mg/dl) (<5.0 : 5.0≥)	58 : 53	41 : 47	0.52
Lymphocyte Count (/μl) (<500 : 500-799 : ≥800)	26 : 31 : 54	36 : 26 : 26	0.006*
Cause of intubation (Head and Neck : Chest and Abdomen : others)	38 : 48 : 33	15 : 56 : 17	0.004*
Intubation period (days) (≤7 : 8-14 : ≥15)	41 : 36 : 34	25 : 32 : 31	0.39
Surgical method (ST : PDT)	69 : 42	53 : 35	0.72

MV: mechanical ventilation; PDT: percutaneous dilatational tracheostomy; ST: surgical tracheostomy.

**Table 4.** Multivariable Analysis of Factors Associated with MV Withdrawal.

		Univariable				Multivariable			
		coef	OR	95%CI	P value	coef	OR	95%CI	P value
Age (year)	20-39	reference				reference			
	40-64	-0.08	0.923	0.205-4.153	0.917	0.943	2.568	0.413-15.595	0.312
	65-74	-1.03	0.357	0.084-1.527	0.165	0.023	1.023	0.170-6.156	0.98
	≥75	-0.653	0.520	0.127-2.138	0.365	0.470	1.6	0.276-9.269	0.6
Sex	Male	-0.446	0.64	0.349-1.175	0.150	-0.032	0.969	0.428-2.195	0.939
BMI	18.5≤ <25	reference				reference			
	<18.5	-0.174	0.840	0.434-1.627	0.605	-0.14	0.869	0.403-1.875	0.72
	25≤ <30	-0.134	0.875	0.372-2.058	0.760	-0.581	0.559	0.208-1.505	0.25
	≥30	-0.762	0.467	0.154-1.410	0.177	-1.784	0.168	0.044-0.634	**0.009＊**
Smoking	Smoker	-0.8	0.449	0.252-0.802	**0.007＊**	-0.616	0.54	0.247-1.182	0.123
Albumin	≤1.9	reference				reference			
	2.0-2.4	0.158	1.171	0.617-2.224	0.629	-0.19	0.827	0.380-1.798	0.631
	≥2.5	0.972	2.643	1.220-5.726	0.014	0.799	2.224	0.919-5.380	0.076
CRP	<5.0	reference				reference			
	≥5.0	-0.227	0.797	0.455-1.396	0.428	0.227	1.255	0.619-2.546	0.529
Lymphocyte count	<500	reference				reference			
	500-799	0.501	1.651	0.799-3.410	0.176	0.69	1.995	0.886-4.493	0.096
	≥800	1.056	2.876	1.445-5.722	**0.003＊**	1.218	3.382	1.497-7.643	**0.003＊**
Cause of intubation	Chest and Abdomen	reference				reference			
	Head and Neck	1.084	2.956	1.451-6.019	**0.003＊**	1.592	4.911	1.741-13.856	**0.003＊**
	Others	0.54	1.716	0.829-3.549	0.145	0.262	1.299	0.538-3.135	0.56
Intubation period	≤7	reference				reference			
	8‐14	-0.377	0.686	0.345-1.366	0.283	-0.394	0.674	0.306-1.484	0.327
	≥15	-0.402	0.669	0.333-1.341	0.257	-0.225	0.798	0.323-1.973	0.626
Surgical method	ST	reference				reference			
	PDT	-0.081	0.922	0.519-1.636	0.781	0.972	2.643	0.890-7.854	0.08

CI: confidence interval; coef: coefficient; MV: mechanical ventilation; OR: odds ratio; PDT: percutaneous dilatational tracheostomy; ST: surgical tracheostomy.

## Discussion

The advantages of tracheostomy for patients undergoing long-term intubation include the elimination of the need for sedation associated with oral intubation and easier management of airway secretions. However, the eventual goal is discharge to home. To achieve this goal, it is necessary to wean the patient off MV. The rates of weaning from MV after tracheostomy in long-term intubated patients range from 47.9% to 70.8% ^(4), (5), (6), (7)^. Cannulation is preferred for the management of cannulas at home. In this study, we focused on weaning from MV and decannulation during the discharge process and examined the factors involved.

Positive ^(6), (8), (9)^ and negative ^(10)^ results have been reported for early tracheostomy after intubation. Morakami et al. ^(9)^ reported that early tracheostomy shortens the duration of MV and improves survival rates. Hsu et al. ^(6)^ reported that tracheostomy >3 weeks may lead to increased MV weaning difficulties and ICU mortality. Since tracheostomy is an invasive procedure, it poses a greater burden on patients with poor general health. Therefore, it is crucial to carefully assess the patient’s overall condition and determine the optimal timing for the procedure. Damuth et al. ^(11)^ reviewed the prognosis of tracheostomies in patients undergoing long-term intubation. They reported that 29% (26%-32%) of patients were discharged due to death and 22% (19%-25%) were discharged home. In the present study, 37.7% of the patients died, 51.8% were transferred to another hospital, and 10.6% were discharged home, reflecting a higher rate of death and lower rate of discharge to home relative to Demuth’s study. These factors can vary depending on the scale and function of hospitals. When examining outcomes at discharge, 81.3% of deaths were associated with difficulty weaning off MV, whereas 61.9% of home discharges were linked to successful decannulation after weaning. From the results (Figure 2 and 3), we consider that the short hospital stay in patients with MV weaning difficulties is associated with the high number of discharges due to death in this group. Additionally, patients who faced difficulties with cannulation were more likely to be transferred to other hospitals for cannula management, which made home discharge more difficult. Our findings confirm that decannulation is crucial for home discharge. Therefore, investigating the factors that determine the feasibility of decannulation may contribute to increasing the number of patients eligible for discharge to home.

Various reports have described the causes of MV weaning difficulties in patients undergoing tracheostomies. A review of 1,307 cases by Wu et al. ^(7)^ identified that prolonged hospital stay, elevated blood urea nitrogen levels, low Glasgow Coma Scale scores, low Alb levels, and low inspiratory pressure at the residual volume were factors that made weaning from MV difficult. In the present study, 3 independent factors were identified: low blood lymphocyte count, chest and abdominal diseases, and BMI ≥30 . This is attributed to the frequent impairment of cardiopulmonary function, including that of the lower respiratory tract, in patients with chest diseases. However, the wide 95% confidence interval (CI) for the cause of intubation suggests that it is an unstable factor. Additionally, it is well known that respiratory failure tends to be more severe in patients with a high BMI due to anatomical and respiratory physiological changes, making withdrawal from MV more challenging ^(12)^. All other factors imply a poor general condition. When weaning from MV is difficult, it not only hinders discharge to home but may also make it impossible to enhance the patient’s quality of life. Therefore, the possibility of MV withdrawal difficulties and need for continuous care should be thoroughly explained preoperatively to patients undergoing tracheostomy who present with these factors. Furthermore, no significant differences were observed in the method of tracheostomy (PDT or ST) as a factor influencing the success of weaning from MV. Considering reports that short-term and long-term complications do not differ between ST and PDT ^(13)^, either surgical approach can be considered appropriate. However, various contraindications to PDT should be considered ^(14)^. In cases where technical difficulties or failure of the procedure occur, conversion to an open surgical approach may be required. Consequently, the availability of technical expertise within the intensive care setting is essential. It should be determined based on the surgeon’s proficiency and the presence of contraindications.

Decannulation is the next step for patients who are successfully weaned from MV. Factors reported to hinder decannulation include advanced age, BMI <22, malignant disease, neurological disease, vasopressor use, post-tracheostomy pneumonia, and delirium ^(3), (4)^. In this study, age ≥75 years, head and neck disease, CRP level elevation, and low blood lymphocyte count were identified as independent factors. Decannulation can adversely affect respiratory function due to an increase in dead space. In the elderly, respiratory function is impaired due to decreased respiratory muscle tone associated with prolonged hospitalization, which may be one of the reasons for difficulty in decannulation. Patients with head and neck disease often experience aspiration pneumonia and require cannula placement to prevent aspiration. Elevated CRP levels before tracheostomy suggested inflammation, possibly due to aspiration pneumonia. It has been reported that decannulation is difficult in cases of aspiration pneumonia with CRP elevation ^(15)^. Warnke et al. ^(5)^ reported a poor prognosis in patients with cannulation difficulties. From a rehabilitative standpoint, protocols incorporating respiratory rehabilitation have been shown to facilitate MV weaning and decannulation ^(16)^. Furthermore, Escudero et al. ^(17)^ reported that early swallowing assessments and swallowing rehabilitation significantly reduced the time required for decannulation. Early management of primary and comorbid conditions, along with the initiation of swallowing and respiratory rehabilitation during the initial stages of hospitalization, is necessary for decannulation.

Lymphopenia has been identified as a contributing factor to difficulties in weaning off MV and failure to achieve decannulation. Generally, lymphopenia indicates reduced immunity and increased susceptibility to infection. It is a risk factor for severe disease in COVID-19 ^(18)^ and has been reported to be associated with a poor prognosis in malignant tumors ^(19)^. Lymphopenia is also a useful marker of malnutrition along with serum Alb and total cholesterol levels ^(20)^. Proper nutritional management is crucial for both preoperative and postoperative tracheostomy care. However, the 95% CIs of the multivariate results for lymphocytes are wide for both the factor of MV withdrawal and decannulation. Therefore, it is advisable to accumulate more cases and re-evaluate.

### Conclusion

Long-term tracheal intubation using tracheostomy offers benefits such as facilitating the management of tracheal secretions and weaning patients off oral intubation. The ultimate goal is to enable discharge to home. This study indicated that lymphopenia, chest and abdominal diseases, and a BMI ≥30 were associated with difficulty in weaning from MV. Factors associated with difficulty in decannulation included lymphopenia, age ≥75 years, head and neck disease, and elevated CRP. If the patient receives appropriate treatment for their condition but still requires prolonged intubation and tracheostomy, it is essential to thoroughly explain the potential difficulties of MV withdrawal and decannulation, particularly for those with characteristics aligning with the findings of this study.

Our findings have limitations. Due to the retrospective design of the study, respiratory function, which may affect weaning from MV and decannulation, was not measured before tracheostomy. In addition, the general condition before and after tracheostomy was managed by each department, which may have affected the duration of weaning from MV, decannulation, and the length of hospital stay. Since this study only covers the years from 2014 to 2019, the results were not affected by the COVID-19 pandemic. However, if the study had been conducted during the pandemic, then the results could have been influenced by its effects.

## Article Information

### Conflicts of Interest

None

### Author Contributions

Ichita Kinoshita, Masaaki Higashino, Masataka Taniuchi, Tetsuya Terada, Takeshi Tochizawa, Ryo Kawata, and Shin-ichi Haginomori conceived and designed research; Masaaki Higashino and Masataka Taniuchi performed research; Masaaki Higashino, and Takeshi Tochizawa analyzed the data; Ichita Kinoshita, Masaaki Higashino, Tetsuya Terada, Ryo Kawata, and Shin-ichi Haginomori wrote the paper. All authors have read and agreed to the published version of the manuscript.

### Approval by Institutional Review Board (IRB)

The protocols of this study were approved by the Research Ethics Committee (Protocol number: 2021-056) and the Human Studies Committee of Osaka Medical and Pharmaceutical University, Takatsuki City, Osaka, Japan, and in accordance with the tenets set forth in the Declaration of Helsinki.
